# Development and validation of qualitative SYBR®Green Real-Time PCR for detection and discrimination of *Listeria* spp. and *Listeria monocytogenes*

**DOI:** 10.1007/s00253-012-4477-2

**Published:** 2012-10-20

**Authors:** Elodie Barbau-Piednoir, Nadine Botteldoorn, Marc Yde, Jacques Mahillon, Nancy H. Roosens

**Affiliations:** 1 Scientific Institute of Public Health, J. Wytsmanstraat 14, 1050 Brussels, Belgium; 2 Faculty of Bioscience Engineering, Earth and Life Institute, Université Catholique de Louvain, Croix du Sud 2, bte L7.05.12, 1348 Louvain-la-Neuve, Belgium

**Keywords:** Real-time PCR, SYBR®Green, Foodborne pathogens, Detection, *Listeria*, qPCR validation

## Abstract

A combination of four qualitative SYBR®Green qPCR screening assays targeting two levels of discrimination: *Listeria* genus (except *Listeria grayi*) and *Listeria monocytogenes*, is presented. These assays have been developed to be run simultaneously using the same polymerase chain reaction (PCR) programme. The paper also proposes a new validation procedure to specifically validate qPCR assays applied to food microbiology according to two guidelines: the ISO 22118 norm and the “Definition of minimum performance requirements for analytical methods of GMO testing”. The developed assays target the *iap*, *prs* and *hlyA* genes that belong to or neighbour the virulence cluster of *Listeria* spp. The selected primers were designed to amplify short fragments (60 to 103 bp) in order to obtain optimal PCR efficiency (between 97 and 107 % efficiency). The limit of detection of the SYBR®Green qPCR assays is two to five copies of target genes per qPCR reaction. These assays are highly accurate (98.08 and 100 % accuracy for the *Listeria* spp. and *L. monocytogenes* assays, respectively).

## Introduction

*Listeria* are small Gram-positive bacilli, ubiquitous, non-sporeforming, facultative anaerobic bacteria that grow between −2 and 50 °C, with optimal growth between 30 and 37 °C (Bajard et al. 1996; Farber and Peterkin 1991). The *Listeria* genus officially includes six species: *Listeria monocytogenes*, *Listeria ivanovii*, *Listeria innocua*, *Listeria seeligeri*, *Listeria welshimeri*, *Listeria grayi* (Garrity et al. 2004). Two other species, *Listeria marthii* (close to *L. monocytogenes* and *L. innocua*) and *Listeria rocourtiae* (close to *L. grayi*), have recently been described (Graves et al. 2010 and Leclercq et al. 2010) but have not yet been introduced into the official classification (Garrity et al. 2004). Among those, *L. monocytogenes* is the most reported as pathogenic for humans (listeriosis) (McLauchlin et al. 2004). However, some cases of listeriosis have also been attributed to *L. ivanovii* (Cummins et al. 1994; Guillet et al. 2010; Lessing et al. 1994), *L. innocua* (Perrin et al. 2003) and *L. seeligeri* (Rocourt et al. 1986).

*L. monocytogenes* has a low annual incidence in Europe, with about 1,500 cases per year (Anonymous 2011a), but with its high fatality rate listeriosis ranks among the most frequent cause of human death due to foodborne illnesses (Cardoen et al. 2009; de Valk 2005). Foodborne listeriosis mainly affects a specific group of the population with increased susceptibility: the YOPI (young, old, pregnant and immunodepressed) (Anonymous 2001). Hence, due to ageing of the population, control of *Listeria* spp. is becoming an increasingly important issue.

Listeriosis cases are associated with the consumption of raw food products (Berrada et al. 2006; Tham et al. 2000; Inoue et al. 2000; Rocourt et al. 2000; de Valk 2001; Inoue et al. 2000), ready-to-eat food (de Valk 2001) and post-processing contaminated food (Maijala et al. 2001; Makino et al. 2005; Norrung et al. 1999). The ability of *Listeria* spp. to grow at low temperatures increases the risk of infection. International standards exist for the detection and enumeration of *L. monocytogenes* in food and feed (Anonymous 1996a; Anonymous 1996b). These methods are time consuming (at least 5 days) and labour intensive. In order to quickly identify the source of foodborne outbreaks or for a faster commercial batch release, tools for rapid detection and identification of pathogens in food have been developed. The majority of these assays are limited to the specific detection of *L. monocytogenes*. They are classical PCR assays (Amagliani et al. 2004; Jung et al. 2003; Li et al. 2000; Liu et al. 2004; Mukhopadhyay and Mukhopadhyay 2007; Winters et al. 1999), reverse-transcription PCR (RT-PCR) (Klein and Juneja 1997) and, more recently, real-time PCR (qPCR) using mainly the TaqMan® technology (Hough et al. 2002; O’Grady et al. 2008; Oravcova et al. 2007; Rossmanith et al. 2006; Rudi et al. 2005). They are targeting genes such as *iap*, *prfA* and *hlyA* involved in *L. monocytogenes* pathogenicity (Dussurget et al. 2004) and are therefore specific for this species.

In this paper, we propose a new detection system that facilitates a rational detection of pathogenic bacteria using as study case the *Listeria* genus, considered as one of the most important of foodborne pathogens transmitted by food and water (Cardoen et al. 2009; Anonymous 2011b). The Combinatory SYBR®Green qPCR screening for foodborne pathogens (CoSYPS Path Food) is based on two detection levels. A first set of generic assays allows the detection of the presence of all bacteria belonging to the *Listeria* genus*.* A second set of assays allows the specific detection of *L. monocytogenes*. The four primer pairs were chosen in order to perform at the same PCR conditions, allowing the different assays to be performed as four simplex assays simultaneously, on the same plate. Moreover, as there is no official method to validate a qPCR assay applied to food microbiology, a guideline to validate food microbiology qPCR assay based on the ISO 22118 norm (Anonymous 2011b) and the “Definition of minimum performance requirements for analyticals methods of GMO testing” (Anonymous 2008a) is also proposed. The four SYBR®Green qPCR assays developed in this study were evaluated for selectivity, sensitivity, dynamic range, PCR efficiencies, repeatability and reproducibility. The advantages of the CoSYPS Path Food screening strategy and the validation guideline are discussed.

## Materials and methods

### Bacterial strains

The bacterial strains used in this study are listed in Table 1. A panel of 128 bacterial, two mold, two yeast and two virus strains has been used. The strains were obtained from the National Reference Centres and Laboratories.

**Table 1:** Selectivity assessment of the four SYBR®Green qPCR assays: “*iap*-50-deg”, “*prs*-2-deg”, “*hlyA*-177” and “*hlyA*-146-deg-tronc”

Genus	Species	Serogroup	GRAM	Origin	Reference	*iap*-50-deg	p*r*s*-*2-deg	*hlyA-*177	*hlyA*-146-deg-tronc
*Listeria*	*monocytogenes*	1/2a	+	List-NRC	10/13	+	+	+	+
*Listeria*	*monocytogenes*	1/2a	+	List-NRC	10/14	+	+	+	+
*Listeria*	*monocytogenes*	1/2a	+	List-NRC	10/15	nt	nt	+	+
*Listeria*	*monocytogenes*	1/2a	+	List-NRC	10/20	nt	nt	+	+
*Listeria*	*monocytogenes*	1/2a	+	List-NRC	10/21	nt	nt	+	+
*Listeria*	*monocytogenes*	1/2a	+	List-NRC	10/22	nt	nt	+	+
*Listeria*	*monocytogenes*	1/2a	+	List-NRC	10/23	nt	nt	+	+
*Listeria*	*monocytogenes*	1/2a	+	List-NRC	ATCC 51772	+	+	+	+
*Listeria*	*monocytogenes*	1/2b	+	List-NRC	ATCC 51777	+	+	+	+
*Listeria*	*monocytogenes*	1/2b	+	List-NRC	10/2	+	+	+	+
*Listeria*	*monocytogenes*	1/2b	+	List-NRC	10/28	+	+	+	+
*Listeria*	*monocytogenes*	1/2b	+	List-NRC	10/50	nt	nt	+	+
*Listeria*	*monocytogenes*	1/2b	+	List-NRC	10/68	nt	nt	+	+
*Listeria*	*monocytogenes*	1/2b	+	List-NRC	10/109	nt	nt	+	+
*Listeria*	*monocytogenes*	1/2c	+	List-NRC	10/48	+	+	+	+
*Listeria*	*monocytogenes*	1/2c	+	List-NRC	10/16	nt	nt	+	+
*Listeria*	*monocytogenes*	1/2c	+	List-NRC	10/49	nt	nt	+	+
*Listeria*	*monocytogenes*	1/2c	+	List-NRC	10/58	nt	nt	+	+
*Listeria*	*monocytogenes*	1/2c	+	List-NRC	10/153	nt	nt	+	+
*Listeria*	*monocytogenes*	1/2c	+	List-NRC	10/160	nt	nt	+	+
*Listeria*	*monocytogenes*	1/2c	+	List-NRC	10/184	nt	nt	+	+
*Listeria*	*monocytogenes*	1/2c	+	List-NRC	10/192	nt	nt	+	+
*Listeria*	*monocytogenes*	3a	+	List-NRC	10/29	+	+	+	+
*Listeria*	*monocytogenes*	3a	+	List-NRC	10/202	nt	nt	+	+
*Listeria*	*monocytogenes*	3a	+	List-NRC	10/237	nt	nt	+	+
*Listeria*	*monocytogenes*	3a	+	List-NRC	9/109	nt	nt	+	+
*Listeria*	*monocytogenes*	3a	+	List-NRC	9/181	nt	nt	+	+
*Listeria*	*monocytogenes*	3a	+	List-NRC	8/171	nt	nt	+	+
*Listeria*	*monocytogenes*	3b	+	List-NRC	8/115	+	+	+	+
*Listeria*	*monocytogenes*	3b	+	List-NRC	Würzburg	+	+	+	+
*Listeria*	*monocytogenes*	3c	+	List-NRC	6/64	+	+	+	+
*Listeria*	*monocytogenes*	3c	+	List-NRC	6/125	+	+	+	+
*Listeria*	*monocytogenes*	3c	+	List-NRC	6/137	nt	nt	+	+
*Listeria*	*monocytogenes*	3c	+	List-NRC	6/275	nt	nt	+	+
*Listeria*	*monocytogenes*	3c	+	List-NRC	6/301	nt	nt	+	+
*Listeria*	*monocytogenes*	4a	+	List-NRC	10/118	+	+	+	+
*Listeria*	*monocytogenes*	4a	+	List-NRC	ATCC 19114	nt	nt	+	+
*Listeria*	*monocytogenes*	4b	+	List-NRC	ATCC 51780	+	+	+	+
*Listeria*	*monocytogenes*	4b	+	List-NRC	10/7	+	+	+	+
*Listeria*	*monocytogenes*	4b	+	List-NRC	10/1	nt	nt	+	+
*Listeria*	*monocytogenes*	4b	+	List-NRC	10/3	nt	nt	+	+
*Listeria*	*monocytogenes*	4b	+	List-NRC	10/24	nt	nt	+	+
*Listeria*	*monocytogenes*	4b	+	List-NRC	10/34	nt	nt	+	+
*Listeria*	*monocytogenes*	4b	+	List-NRC	10/47	nt	nt	+	+
*Listeria*	*monocytogenes*	4c	+	EU-RL List	09LEB41LM	+	+	+	+
*Listeria*	*monocytogenes*	4d	+	List-NRC	8/221	+	+	+	+
*Listeria*	*monocytogenes*	4d	+	List-NRC	7/89	nt	nt	+	+
*Listeria*	*monocytogenes*	4d	+	List-NRC	7/114	nt	nt	+	+
*Listeria*	*monocytogenes*	4d	+	List-NRC	5/163	nt	nt	+	+
*Listeria*	*monocytogenes*	4e	+	List-NRC	10/35	+	+	+	+
*Listeria*	*ivanovii*		+	List-NRC	CIP 7842	+	+	−	−
*Listeria*	*ivanovii*		+	ILVO	LMG11388	+	+	−	−
*Listeria*	*ivanovii*		+	List-NRC	06/124	+	+	−	−
*Listeria*	*ivanovii*		+	List-NRC	06/129	+	+	−	−
*Listeria*	*ivanovii*		+	EU-RL List	TQA237	+	+	−	−
*Listeria*	*ivanovii*		+	EU-RL List	TQA238	+	+	−	−
*Listeria*	*ivanovii*		+	EU-RL List	00CHPL02	+	+	−	−
*Listeria*	*seeligeri*		+	List-NRC	ATCC 35967	+	+	−	−
*Listeria*	*seeligeri*		+	ILVO	MB43:LMG16764	+	+	−	−
*Listeria*	*seeligeri*		+	EU-RL List	TQA231	+	+	−	−
*Listeria*	*seeligeri*		+	EU-RL List	TQA232	+	+	−	−
*Listeria*	*welshimeri*		+	List-NRC	ATCC 35897	+	+	−	−
*Listeria*	*welshimeri*		+	List-NRC	06/102	+	+	−	−
*Listeria*	*welshimeri*		+	List-NRC	06/229	+	+	nt	nt
*Listeria*	*welshimeri*		+	List-NRC	04/341	+	+	nt	nt
*Listeria*	*welshimeri*		+	EU-RL List	02CHPL153	+	+	nt	nt
*Listeria*	*welshimeri*		+	EU-RL List	02CHPL154	+	+	nt	nt
*Listeria*	*welshimeri*		+	EU-RL List	03CHPL91	+	+	nt	nt
*Listeria*	*welshimeri*		+	EU-RL List	TQA230	+	+	nt	nt
*Listeria*	*innocua*		+	EU-RL List	03CHPL98	+	+	nt	nt
*Listeria*	*innocua*		+	List-NRC	CIP 8011	+	+	−	−
*Listeria*	*innocua*		+	ILVO	MB176(T) = FML2011	+	+	−	−
*Listeria*	*innocua*		+	List-NRC	10/85	+	+	nt	nt
*Listeria*	*innocua*		+	List-NRC	10/101	+	+	nt	nt
*Listeria*	*innocua*		+	List-NRC	10/122	+	+	nt	nt
*Listeria*	*innocua*		+	List-NRC	09/158	+	+	nt	nt
*Listeria*	*innocua*		+	List-NRC	09/221	+	+	nt	nt
*Listeria*	*innocua*		+	List-NRC	09/291	+	+	nt	nt
*Listeria*	*innocua*		+	List-NRC	08/147	+	+	nt	nt
*Listeria*	*innocua*		+	List-NRC	07/92	+	+	nt	nt
*Listeria*	*innocua*		+	List-NRC	06/237	+	+	nt	nt
*Listeria*	*innocua*		+	IPH-FP	TIAC 706	+	+	nt	nt
*Listeria*	*grayi*		+	List-NRC	ATCC 25401	−	−	−	−
*Listeria*	*grayi*		+	ILVO	LMG16490	−	−	−	−
*Aeromonas*	*hydrophila*		−	IPH-CB	6688 (M/2862 (EEQ 2003/2))	−	−	−	−
*Bacillus*	*cereus*		+	IPH-FP	ATCC 14579	−	−	−	−
*Bacillus*	*circulans*		+	IPH-FP	TIAC 100	−	−	−	−
*Bacillus*	*lentus*		+	IPH-FP	TIAC 101	−	−	−	−
*Bacillus*	*lichiniformis*		+	IPH-FP	TIAC 102	−	−	−	−
*Bacillus*	*mycoides*		+	IPH-FP	TIAC 97	−	−	−	−
*Bacillus*	*sphaericus*		+	IPH-FP	TIAC 104	−	−	−	−
*Bacillus*	*subtillis*		+	IPH-FP	TIAC 103	−	−	−	−
*Bacillus*	*thuringiensis*		+	IPH-FP	TIAC 96	−	−	−	−
*Brevibacillus*	*borstelensis*		+	IPH-FP	TIAC 099	−	−	−	−
*Brochothrix*	*thermosphacta*		+	IPH-FP	TIAC 400	−	−	−	−
*Campylobacter*	*coli*		−	IPH-FP	ATCC 33559 T	−	−	−	−
*Campylobacter*	*jejuni*		−	IPH-FP	ATCC 33291	−	−	−	−
*Campylobacter*	*lari*		−	IPH-FP	TIAC 542	−	−	−	−
*Carnobacterium*	*divergens*		+	IPH-FP	Argentijns vlees B21	−	−	−	−
*Citrobacter*	*freundii*		−	IPH-FP	TIAC 554	−	−	−	−
*Clostridium*	*perfingens*		+	IPH-FP	ATCC 13124 T	−	−	−	−
*Enterobacter*	*cloacae*		−	IPH-FP	TIAC 445	−	−	−	−
*Enterococcus*	*faecalis*		+	IPH-CB	ATCC 29212	−	−	−	−
*Escherichia*	*coli*		−	IPH-FP	ATCC 25922	−	−	−	−
*Escherichia*	*coli*	O157	−	IPH-FP	EH 630	−	−	−	−
*Hafnia*	*alvei*		−	IPH-CB	7186	−	−	−	−
*Klebsiella*	*pneumoniae*		−	IPH-FP	TIAC 446	−	−	−	−
*Lactobacillus*	*acidophilus*		+	IPH-FP	Argentijns vlees A19	−	−	−	−
*Lactobacillus*	*brevis*		+	IPH-FP	Argentijns vlees A53	−	−	−	−
*Lactobacillus*	*curvatus*		+	IPH-FP	Argentijns vlees A1	−	−	−	−
*Lactobacillus*	*delbrucki*		+	IPH-FP	Argentijns vlees B17	−	−	−	−
*Lactobacillus*	*plantarum*		+	IPH-FP	Argentijns vlees B34	−	−	−	−
*Lactococcus*	*lactis lactis*		+	IPH-FP	Argentijns vlees A31	−	−	−	−
*Leuconostoc*	*citreum*		+	IPH-FP	Argentijns vlees B24	−	−	−	−
*Leuconostoc*	*mesenteroides*		+	IPH-FP	Argentijns vlees B6	−	−	−	−
*Paenibacillus*	*polymyxa*		+	IPH-FP	TIAC 105	−	−	−	−
*Proteus*	*vulgaris*		−	IPH-CB	6223 (M/654) (EEQ 1996/3)	−	−	−	−
*Pseudomonas*	*aeruginosa*		−	IPH-FP	LMG 6395	−	−	−	−
*Salmonella*	*enterica enterica*	Enteritidis	−	Salm-NRC	H, VI, 6, 32	−	−	−	−
*Salmonella*	*enterica enterica*	Thyphimurium	−	Salm-NRC	H, II, 32, 32	−	−	−	−
*Serratia*	*marcescens*		−	IPH-CB	7015	−	−	−	−
*Shigella*	*sonneï*		−	Salm-NRC	10-03865	−	−	−	−
*Staphylococcus*	*aureus*		+	IPH-FP	ATCC 25923	−	−	−	−
*Staphylococcus*	*epidermidis*		+	IPH-FP	TIAC 367	−	−	−	−
*Staphylococcus*	*pisciferm*		+	IPH-FP	TIAC 364	−	−	−	−
*Streptococcus*	*feacales*		+	IPH-FP	TIAC 300	−	−	−	−
*Vibrio*	*parahaemoliticus*		−	IPH-FP	TIAC 610	−	−	−	−
*Yersinia*	*enterocolitica*		−	IPH-FP	LMG 15558	−	−	−	−
*Aspergillus*	*fumigatus*		na	IPH-MA	BCCM/IHEM 19436	−	−	−	−
*Cladosporium*	*sphaerospermum*		na	IPH-MA	BCCM/IHEM 24474	−	−	−	−
*Saccharomyces*	*cerevisiae*		na	IPH-MA	BCCM/IHEM 3961	−	−	−	−
*Candida*	*parapsilosis*		na	IPH-MA	BCCM/IHEM 6478	−	−	−	−
Hepatitis *A Virus*			na	IPH-FP	27 (WZ)	−	−	−	−
Norovirus			na	IPH-FP	2593	−	−	−	−
No template control				na	na	−	−	−	−

*+* there is an amplification and a *T*_m_ value similar for all corresponding strains, − no amplification, *List-NRC* Belgian *Listeria* National Reference Centre, rue Juliette Wytsmanstraat 14, 1050 Brussels, Belgium, *EU-RL List* EU-RL *Listeria monocytogenes*, 23 avenue du Général de Gaulle, 94706 Maisons-Alfort cedex, France, *ILVO* Instituut voor Landbouw- en Visserijonderzoek, Technology & Food Science Unit, Food Safety, Product Quality and Innovation, and Business Unit and Service Centre, Brusselsesteenweg 370, 9090 Melle, Belgium, *IPH-FP* Scientific Institute of Public Health, Food Pathogens Laboratory, rue Juliette Wytsmanstraat 14, 1050 Brussels, Belgium, *IPH-CB* Scientific Institute of Public Health, Clinical Biology, rue Juliette Wytsmanstraat 14, 1050 Brussels, Belgium, *Salm-NRC* Belgian *Salmonella* and *Shigella* National Reference Centre, rue Juliette Wytsmanstraat 14, 1050 Brussels, Belgium, *IPH-MA* Mycology and Aerobiology, Scientific Institute of Public Health, rue Juliette Wytsmanstraat 14, 1050 Brussels, Belgium, *nt* not tested, *na* not applicable

### Bacterial growth conditions, DNA extraction and DNA quantification

Overnight cultures of each bacterial strain were grown in liquid brain–heart infusion or Bolton liquid medium (for *Campylobacter*) at adequate temperature and oxygen conditions. The total DNA from each strain was extracted with the DNeasy Blood and Tissue Kit (Qiagen). Genomic DNA (gDNA) from yeast and fungal were extracted using the Invisorb Spin Plant Mini Kit (STRATEC Molecular GmbH) and ZR Fungal/Bacterial gDNA extraction (Zymo Research), respectively. Viral RNA was extracted using the RNeasy mini kit (Qiagen) and cDNA was obtained by reverse transcription using the Transcriptor high-fidelity cDNA synthesis kit (Roche). cDNA amplification was checked with a specific PCR amplification. All kits were used according to the manufacturer’s recommendations. DNA quality was controlled on agarose gel and DNA concentration was measured using a Nanodrop® 2000 device according to the manufacturer’s recommendations.

### Development and *in silico* assessment of primer pairs

A uniform primer design approach was applied in the development of all primer pairs. The first step consisted of collecting a set of genes of potential interest, either genus or species specific (Glaser et al. 2001; Hough et al. 2002; Kerouanton et al. 2009; Liu et al. 2004; McLauchlin et al. 2004; O’Grady et al. 2008; Oravcova et al. 2007; Pan and Breidt 2007). The second step included the collection of DNA sequences relevant for the selected targets from the NCBI public database (http://www.ncbi.nlm.nih.gov/sites/entrez). The primer pairs were designed, preferentially within conserved regions, using the “Primer 3” programme (http://frodo.wi.mit.edu/primer3/) (Rozen and Skaletsky 2000) with the “product size range” specification set at “60 to 120 bp” and “primer size” optimal set at “22 bases”. An *in silico* test of the primer pairs' selectivity was then performed. This test consisted of a bioinformatical analysis carried out with the “wprimersearch” software (https://wemboss.uio.no/wEMBOSS/) (Rice et al. 2000; Sarachu and Colet 2005), which mimics the PCR amplification of the tested primers on a database of bacterial genome sequences from NCBI of 217 bacteria, representing 103 species belonging to 61 genera. Only primer pairs that gave the expected *in silico* amplification were retained for the *in situ* test. When mismatches between the primers and one of the targets were observed, degenerate nucleotides were introduced into the primer sequence. However, primer pairs with no degenerate nucleotides were always preferred.

### Qualitative SYBR®Green qPCR assay

All qPCR assays were performed in accordance with the general requirements from the ISO norm 22119 (Anonymous 2011c) except those specific for the TaqMan® chemistry since the SYBR®Green was used. All qPCR assays were performed on an Applied Biosystems 7300 Real-Time PCR System (Applied Biosystems) with MicroAmp® Optical 96-Well Reaction Plate closed with the MicroAmp® Optical 8-Cap Strip (Applied Biosystems). The reaction was performed in a final volume of 25 μl containing 5 μl of the appropriate template (10^4^ copies of gDNA for the selectivity test or serial dilution of gDNA for the sensitivity test), 1X SYBR®Green PCR Mastermix (Diagenode) and the appropriate concentration of each primer (Table 2). The following thermal programme was applied: a single cycle of DNA polymerase activation for 10 min at 95 °C followed by 40 amplification cycles of 15 s at 95 °C (denaturing step) and 1 min at 60 °C (annealing & extension step). Subsequently, melting temperature analysis of the amplification products was performed by gradually increasing the temperature from 60 to 95 °C in 20 min (± 0.6 °C/20s). The fluorescent reporter signal was normalized against the internal reference dye (ROX) signal and the threshold limit setting was performed in automatic mode, according to the ABI Sequence Detection Software version 1.4 (Applied Biosystems). “No template” controls (NTC) using DNase and RNase free water (Acros) were included in each reaction to assess primer dimer formation or non-specific amplification.

**Table 2:** Primer pair sequence, concentration and amplicon size and Tm value for each SYBR®Green qPCR assay

Targeted genus	Targeted species	Targeted gene	Primer pair name	Primer name	Primer sequence, 5′→3′	Primer concentration (nM)	Product size (bp)	*T*_m_ amplicon	Reference
*Listeria*	All (except *L. grayi)*	Invasion-associated protein (*iap*)	*iap*-50-deg	*iap*-31-deg-F	CA*Y*CCGC*W*AGCAC*W*GTAGTAGT	1,000	78	75.5–76 °C *L. monocytogenes*, *L. seeligeri*, *L. welshimeri* and *L. ivanovii*; 77 °C *L. innocua*	This study
				*iap*-50-deg-R	GCGTC*R*ACAGT*W*GT*S*CC*H*TT	1,000			
		Phosphoribosyl pyrophosphate synthetase (*prs*)	*prs*-2-deg	*prs*-2-F	ATTTTCTCGCTAAATTCTAATCGTG	250	60	71.5 °C	This study
				*prs*-2-R-deg	CAATACC*W*ACTTCTTTCGCAATCTC	250			
*Listeria*	*monocytogenes*	Listeriolysin O (*hlyA*)	*hlyA*-177	*hlyA*-177-F	TGCAAGTCCTAAGACGCCA	250	112	74 °C	Nogva et al. (2000)
				*hlyA*-177-R	CACTGCATCTCCGTGGTATACTAA	250			
			*hlyA*-146-deg-tronc	*hlyA*-146-deg-tronc-F	AAATCTGTCTCAGG*Y*GATGT	1,000	103	73.5–74 °C for *L. monocytogenes* 1/2a, 1/2c, 3a, 3c, 4a, 4d and 4e and 75 °C for *L. monocytogenes* 1/2b, 3b, 4b and 4c	Adapted from Hough et al. (2002)
				*hlyA*-146-R	CGATGATTTGAACTTCATCTTTTGC	1,000			

In italics are degenerated nucleotides: R = A or G, Y = C or T, W= A or T, S = C or G, H = A or C or T

For the interpretation of a SYBR®Green qPCR assay, two criteria were taken into consideration: the quantification cycle (*C*_q_) value and the melting temperature of the amplicon (*T*_m_). The *C*_q_ value represents the fractional cycle at which PCR amplification reaches the threshold level for the reaction (Bustin 2000). Since it is a screening assay, a qualitative response is required. To be considered as positive, a signal generated in SYBR®Green qPCR analysis should display an (exponential) amplification above the threshold level, with a single peak upon melting analysis giving a unique *T*_m_ value. A signal was considered as negative when no *C*_q_ value was obtained.

### Selectivity test and accuracy calculation

Primer pairs that passed the *in silico* evaluation were tested *in situ*. The latter selectivity test consisted of two steps:

1. A preliminary selectivity test involving few target strains (closely relative families) and few non-target strains (most important pathogenic bacteria) was performed. Primer pairs amplifying only the target strains were tested for full selectivity.
2. The full selectivity test allows testing the inclusivity and exclusivity of each developed assay. This experimental design follows the ISO 22118 norm (Anonymous 2011b) as it involves 50 target strains and at least 52 non-target strains representing 53 species belonging to 29 genera and a NTC (Table 1) (Anonymous 2011b). The non-target relevant microorganisms to test the exclusivity were chosen among taxonomically closely related and not closely related (pathogenic or not) bacteria that can be present in the food matrices (Anonymous 2011b).

The qPCR reactions were performed with approximately 10^4^ copies of genomic DNA calculated according to the genome size of each targeted bacteria using the following formula:

$$ {{C}_{\text{n}}}\frac{{m\times \text{Ac}}}{{{{M}_{\text{w}}}\times {{G}_{\text{s}}}}} $$

where *C*_n_ = copy number, *m* = amount of gDNA (grams), Ac = Avogadro’s constant (Mohr et al. 2008) = 6.02214179 × 10^23^ mol^−1^, *M*_w_ = base pair mean molecular weight = 649 Da and *G*_s_ = Genome size (in base pairs).

The accuracy of the assay can be calculated from the selectivity test. The accuracy represents the closeness of agreement between a test result and the accepted reference value (Anonymous 1993). Its formula is found in Anonymous (2003). Five criteria were set to define a “specific signal” generated in the selectivity of a SYBR®Green qPCR analysis (Barbau-Piednoir et al. 2010): (1) an (exponential) amplification above the threshold level should be obtained with template DNA for the positive strains, while negative controls (NTC and gDNA from negative strains) should not yield such amplification, (2) with positive strain template DNA, the obtained PCR product(s) should present a single peak upon melting analysis with a unique *T*_m_ value, while no specific peak should be detectable in the negative strains and negative controls, (3) positive reactions should display a single band on agarose gel analysis with (4) a size corresponding to the one predicted (SD ± 10 bp) and (5) the sequence of the amplicon, verified by sequencing, should be correct as to guarantee that the amplified fragment is indeed the target.

### Amplicon cloning and sequencing

All PCR reactions for cloning and sequencing were performed on an iCycler PCR System (Biorad) in 25-μl reaction volume containing 10^6^ copies of gDNA, 1 U Pfu DNA Polymerase (Fermentas), 1X DNA polymerase buffer, 0.2 mM of each dNTP and 250 nM of each primer (Table 2; T7 forward and M13 reverse primer). The following thermal programme was applied: a single cycle of initial denaturation for 2 min at 95 °C followed by 30 amplification cycles of 15 s at 95 °C (denaturing step), 15 s at 60 °C (annealing step) and 1 min at 72 °C (extension step). A subsequent final elongation step of the amplification products was performed using 72 °C for 10 min.

PCR fragments obtained by “classical” PCR amplification using *L. monocytogenes* 1/2a strain ATCC 51772 as template were cloned into a TOPO pCR®2.1 plasmid (InVitrogen) according to the manufacturer’s recommendations. The plasmids containing the different amplicons (TOPO pCR® 2.1-amplicon) were transformed into TOP10F’ competent cells (InVitrogen) according to the manufacturer’s recommendations. The “TOPO pCR 2.1-amplicon” was checked for insert length by PCR reaction using T7 forward and M13 reverse primers. Agarose gel electrophoresis was performed using 1 or 3 % precast gels (Biorad) and 1x TBE (89 mM TRIS-borate, 2 mM EDTA) at 100 V for 15 min, including a 100-bp–2-kb Molecular Marker (BioRad). Plasmids that gave the expected size on agarose gel were purified with the Plasmid Mini kit (Qiagen). The inserts were then sequenced with the T7 forward and M13 reverse primers using a dideoxy sequence analysis on an ABI3130xl Genetic Analyzer apparatus (Applied Biosystems) with the BigDye Terminator v3.1 cycle sequencing kit (Applied Biosystems) according to the manufacturer’s recommendations. Forward and reverse sequences of a “TOPO pCR2.1-amplicon” plasmid were aligned by ClustalW2 software (http://www.ebi.ac.uk/Tools/msa/clustalw2/). The amplicon sequence was compared to NCBI sequences database (MEGABLAST) to confirm that it corresponds to the target gene (http://blast.ncbi.nlm.nih.gov/Blast.cgi).

### Determination of the optimal primer concentration

The optimal concentration of the selected primer pairs was determined by testing different concentrations of each primer between 250 and 1,000 nM. The concentrations giving the lowest *C*_q_ value without inducing the formation of a high level of primer dimers were selected. The primer dimer dissociation peak should not be higher than the dissociation peak from the positive samples from high concentrations until the limit of detection (LOD).

### Dynamic range and calculation of the PCR efficiency

The dynamic range of an assay is the concentration range where it performs in a linear manner. The SYBR®Green qPCR assays' dynamic range was assessed by the analysis in duplicate of a serial dilution in a carrier DNA (4 ng/μl calf thymus DNA (Invitrogen)) of pure strain DNA (1,000 to 0.01 theoretical genomic copies) of *L. monocytogenes* 1/2a ATCC 51772 and *L. ivanovii* LMG 11388 for *Listeria* spp. assays and *L. monocytogenes* 1/2a ATCC 51772 and *L. monocytogenes* 4b ATCC 51777 for *L. monocytogenes* assays. The carrier DNA is meant to avoid the dilution problem associated with low gDNA concentration. These analyses allow the assessment of the coefficient of determination (*R* ^2^) and the PCR efficiency (*E*) for each SYBR®Green qPCR assay. *R* ^2^ is an indicator of the correlation of data regarding the linear regression curve. *R* ^2^ of a dynamic range curve should be above 0.98 (Anonymous 2008a). *E* should be between 89.6 and 110.2 % (Anonymous 2008a) and can be calculated according to the formula described in Rutledge and Cote (2003).

### Sensitivity test

Primer pairs passing the selectivity test were subsequently examined for their sensitivity. Using serial dilution, the SYBR®Green qPCR assays were tested to evaluate their LOD, which is defined as the concentration of an analyte that gives a positive result with a probability of 95 % (Anonymous 2008). The strains used were *L. monocytogenes* 1/2a ATCC 51772 and *L. ivanovii* LMG 11388 for *Listeria* spp. assays and *L. monocytogenes* 1/2a ATCC 51772 and *L. monocytogenes* 4b ATCC 51777 for *L. monocytogenes* assays. The calculation of the target genomic copy numbers for each dilution point was done according to the equation given in the selectivity part, considering that the gDNA size of *L. monocytogenes* and *L. ivanovii* are 2,976,163 bp (accession # CP002816) and 2,928,879 bp (accession # FR687253), respectively. To determine LOD, a range of copy number between 10 and 0.1 theoretical copies was tested (i.e. 10, 5, 2, 1, 0.5, 0.2 and 0.1). Each dilution was tested in six replicates per plate for both strains. Moreover, the analysis was performed three independent times under repeatable conditions, resulting in 36 repeats for each dilution point. The dilution series continued after the theoretical single copy to assess the dilution series correctness. Indeed it is statistically impossible to get amplification in all the reactions with the dilutions below 1 theoretical copy. If this is the case, the template concentration has to be checked and the dilution series have to be redone.

### Repeatability calculation

To evaluate the repeatability of the assays, the data from the independent tests performed for the sensitivity test, with the same protocol, with the same samples and by the same operator using the same apparatus within a short interval of time (Anonymous, 1993) were used. The repeatability limit (*r*) is the maximal difference between two test results, obtained under repeatable conditions, expected with a probability of 95 % (Anonymous, 1993). In future analyses, if the difference between values obtained under repeatable conditions exceeds *r*, the values should be considered suspicious. The repeatability limit is obtained with the formula found in Anonymous (2003).

The relative standard deviation of repeatability (RSDr) represents the absolute value of the coefficient of variation. It is expressed in percent and is obtained by multiplying the repeatability standard deviation by 100 and dividing this product by the repeatability median (Anonymous 2003).

According to the guidelines of Anonymous (2008a), RSDr should be ≤ 25 % for all the dilutions above LOD. The RSDr and *r* values of the *C*_q_ values have been calculated at each dilution point. The RSDr and *r* values of the *T*_m_ values have been calculated with all the *T*_m_ values coupled with amplification (*C*_q_ ≠ 40).

### Reproducibility study and calculation

To evaluate the reproducibility of the assays (Anonymous 1993), independent tests were performed with the same protocol, using the same eight samples, in two different laboratories by two different operators using two different apparatus (ABI7300 and Bio-Rad iQ5). The eight samples tested were all gDNA from *L. monocytogenes* strains at different concentrations between 200 and 5 genomic copies per reaction. Each sample was analysed in duplicate by each operator.

Two reproducibility measures can be calculated from these results: the relative standard deviation of reproducibility (RSD_R_) and the uncertainty (*U*). RSD_R_ represents the absolute value of the coefficient of variation. It is expressed in percent and is obtained by multiplying the reproducibility standard deviation by 100 and dividing this product by the reproducibility median (Anonymous 2003).

According to the guidelines of ENGL (Anonymous 2008a), RSD_R_ should be ≤ 35 % for all samples tested. The RSD_R_ of the *C*_q_ and TM values are calculated for the eight samples tested.

“The uncertainty is the parameter associated with the result of a measurement that characterizes the dispersion of the values that could reasonably be attributed to the measurand” (Anonymous 2008b). The uncertainty of the *C*_q_ and *T*_m_ values of each SYBR®Green qPCR assay could be calculated from the results of the reproducibility test. The uncertainty can be expressed by the expanded uncertainty (*U*) which is the quantity defining “an interval about the result of a measurement that may be expected to encompass a large fraction of the distribution of values that could reasonably be attributed to the measurand” (Anonymous 2008b).

The expanded uncertainty *U* is obtained by multiplying the combined standard uncertainty by a coverage factor (Anonymous 2008b).

### Combination of the four SYBR®Green qPCR detection assays

The four assays have been run, on the same plate with the same PCR programme (as described previously), using the appropriate concentration of each primer (Table 2) on 10^4^ copies of four gDNA extractions from pure culture of *L. monocytogenes* 4b (ATCC 51780), *L. ivanovii* (CIP 7848), *L. seeligeri* (ATCC 25401) and *L. grayi* (ATCC 25401). All of these strains were collected from the Belgian National Reference Centre of *L. monocytogenes.*

## Results

### Development and selection of qPCR SYBR®Green assays

#### Selection of the targeted genes

First, a survey of available genetic and genomic data was performed in order to select the *Listeria* genus and *L. monocytogenes* species-specific genes. For the *Listeria* genus, three genes were retained: *iap*, belonging to the known virulence genes of *Listeria* (Kuhn and Goebel 1989), and *ldh* and *prs* that are not involved in the virulence but directly flank a virulence gene cluster (Schmid et al. 2005). These three genes are present in all *Listeria* species (Schmid et al. 2005). For the specific detection of *L. monocytogenes*, the chosen gene was *hlyA*. This gene is involved, and is crucial, in the virulence of *L. monocytogenes* (Dussurget et al. 2004). The four selected genes (*iap*, *prs*, *ldh* and *hlyA*) are present in a single copy on *Listeria* chromosomes (Dussurget et al. 2004; Schmid et al. 2005).

#### Selection of the primer pairs

For the detection of the *Listeria* genus, 50 primer pairs were designed on *iap*, *prs* and *ldh* genes and tested together with another previously described *iap*-based primer pair (Klein and Juneja 1997). For the detection of *L. monocytogenes*, three *hlyA*-based primer pairs were selected from previous works (Hough et al. 2002; Nogva et al. 2000; Thulin Hedberg et al. 2009). After *in silico* evaluation of all these primer pairs and degeneration of some nucleotides (when necessary), 19 primer pairs remained. These 19 primer pairs were tested *in situ* with the preliminary selectivity test (data not shown) and resulted in only six pairs: *iap*-55-deg, *iap*-50-deg, *ldh*-2 and *prs*-2-deg for the *Listeria* spp. detection and *hlyA*-177 and *hlyA*-146-deg-tronc for the *L. monocytogenes* detection. These six pairs were then tested to determine the optimal concentration to be used in the SYBR®Green qPCR amplification (see Table 2). Following this test, the *iap*-55-deg pair was discarded because of the formation of a high level of primer dimers.

### Determination of SYBR®Green qPCR assays selectivity

Table 1 gives an overview of the strains tested for the selectivity test of each qPCR assay. For *iap-*50-deg and *prs-*2-deg *Listeria* spp. detection assays, a specific amplification was observed for 50 of the 52 target strains. The two *L. grayi* strains showed no amplification. As expected, no amplification was observed with the 52 non-target strains and the NTC (Table 1). Thus, these first two assays are 98.08 % accurate for their target (*Listeria* spp.), giving 0 % of false positive and 3.85 % of false negative (two strains of *L. grayi*) results. The *ldh-*2 primer pair assay was discarded during the sensitivity study because of a high level of primer dimers at low target concentrations (data not shown). It has to be mentioned that *L. marthii* (accession # NZ_CM001047.1), which is close to *L. innocua* and *L. monocytogenes* (Graves et al. 2010) can be amplified, *in silico*, by the *Listeria* spp. assays (data not shown) and not by the *L. monocytogenes* assays (data not shown). *L. rocourtiae*, which is closely related to *L. grayi* (Leclercq et al. 2010), will probably be amplified by none of the developed SYBR®Green qPCR assays.

For *hlyA-*177 (Nogva et al. 2000) and *hlyA-*146*-*deg-tronc (adapted from Hough et al. 2002) *L. monocytogenes* detection strategy, a specific amplification was observed with the 50 target strains and no amplification was observed with the 65 non-target strains and the NTC (Table 1). These two approaches are 100 % accurate for their targets (*L. monocytogenes*), giving 0 % of false positive and 0 % of false negative results.

**Fig. 1:**
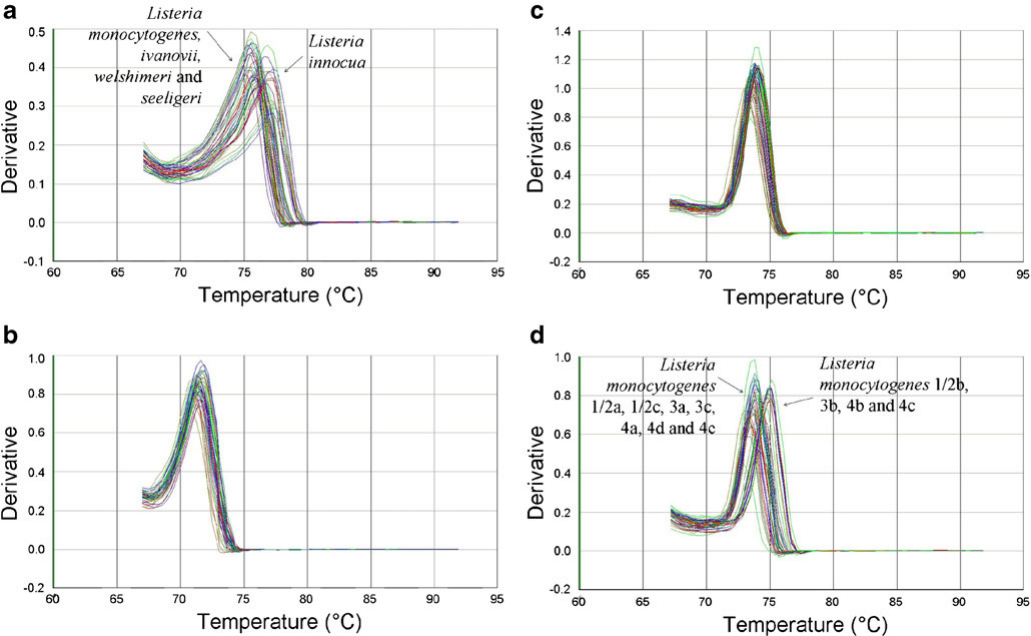
Melting curves obtained by SYBR®Green qPCR analysis of the positive pure strains listed in Table 1. The different qPCR assays are *iap*-50-deg (**a**), *prs*-2-deg (**b**), *hlyA*-177 (**c**) and *hlyA*-146-deg-tronc (**d**). The temperature is plotted on the *X*-axis versus the inverse of the first derivate of the best-fitted curve of the measured fluorescence decrease on the *Y*-axis

These four assays always gave rise to a unique band of the expected size and the sequence of each amplicon corresponded with the one expected of *L. monocytogenes* (data not shown). In addition, all four SYBR®Green qPCR assays gave a unique melting peak for each target with different *T*_m_ values (see Fig. 1 and Table 2).

### Determination of dynamic range and PCR efficiency of SYBR®Green qPCR assays

**Fig. 2:**
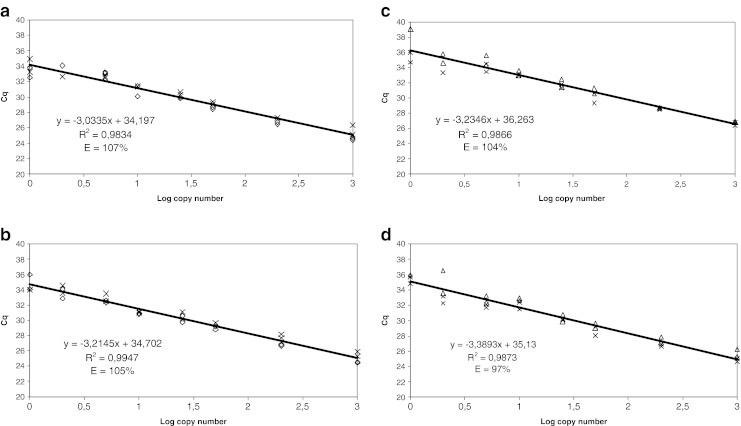
Dynamic range, coefficient of determination and PCR efficiency of the four *Listeria* SYBR®Green qPCR assays methods. *Curves* were obtained from two replicates for each concentration (expressed in copy number) from two different strains. **a** *iap*-50-deg, **b** *prs*-2-deg, **c** *hlyA*-177, **d** *hlyA*-146-deg-tronc. *Multiplication symbol L. monocytogenes* 1/2a (ATCC 51772), *open diamond L. ivanovii* (LMG 11388), *open triangle L. monocytogenes* 4b (ATCC 51777)

All of the assays performed in a linear manner between 1,000 and 1 copy of the targeted gene since the *R* ^2^ values of the four assays, *iap*-50-deg, *prs*-2-deg, *hlyA*-177 and *hlyA*-146-deg-tronc, ranged between 0.9834 and 0.9947 (Fig. 2). The results from the dynamic range analyses allowed the determination of the PCR efficiency (*E*) of each of the four developed SYBR®Green qPCR assays. The four assays displayed a PCR efficiency of 107, 105, 104 and 97 % for *iap*-50-deg, *prs-*2-deg, *hlyA*-177 and *hlyA*-146-deg-tronc, respectively (Fig. 2). The *R* ^2^ and *E* values of the developed SYBR®Green qPCR comply with the acceptance limits.

### Determination of sensitivity and repeatability of SYBR®Green qPCR assays

LOD was identified to be between two to five copies for the four SYBR®Green qPCR assays: *iap*-50-deg, *prs-*2-deg, *hlyA*-177 and *hlyA*-146-deg-tronc (Table 3). The *r* value at LOD of the *C*_q_ values ranges between 1.5 to 4.8 *C*_q_ (Table 4). The *r* value of the *T*_m_ values ranges between 0.5 to 1.3 °C (Table 5). The RSDr value at LOD of the *C*_q_ values was below 5 % for the four developed assays ranging between 1.6 and 4.9 %. The RSDr value of the *T*_m_ values was below 1 % for the four developed assays ranging between 0.2 and 0.6 % (Table 5). The LOD and RSD_r_ values of the developed SYBR®Green qPCR comply with the acceptance limits.

**Table 3:** Limit of detection determination for all *Listeria* SYBR®Green qPCR assays. The results are based on 36 repetitions with genomic targets as DNA template. Average, standard deviation and percentage of positive signal of *C*_q_ values obtained at each dilution point

		Mean *C*_q_ value ± SD (% positive)			
		*Listeria* spp. (except *L. grayi*) assays		*Listeria monocytogenes* assays	
	Theoretical copy number/assay	*iap*-50-deg	*prs*-2-deg	*hlyA*-177	*hlyA*-146-deg-tronc
Serial dilution points	10	32.96 ± 1.02 (100)	31.18 ± 0.74 (100)	32.85 ± 1.12 (100)	33.23 ± 0.87 (100)
	5	34.11 ± 1.45 (97.2)	32.19 ± 0.92 (100)	33.79 ± 1.31 (100)	33.93 ± 0.79 (97.2)
	2	34.75 ± 1.01 (80.6)	33.43 ± 0.70 (72.2)	35.2 ± 1.13 (75)	34.96 ± 1.2 (75)
	1	35.44 ± 1.02 (75)	34.23 ± 1.08 (55.6)	35.63 ± 0.97 (61.1)	34.18 ± 3.78 (50)
	0.5	35.51 ± 0.86 (44.4)	34.67 ± 0.52 (44.4)	36.04 ± 1.20 (36.1)	35.57 ± 1.20 (44.4)
	0.2	35.99 ± 1.05 (19.4)	34.61 ± 0.69 (27.8)	36.08 ± 1.09 (22.2)	35.62 ± 1.69 (22.2)
	0.1	35.85 ± 0.98 (22.2)	34.59 ± 0.48 (16.7)	36.46 ± 0.21 (8.3)	35.64 ± 1.62 (11.1)
Negative controls	Dilution buffer control	Below LOD	Below LOD	Below LOD	Below LOD
	Water control	Below LOD	Below LOD	Below LOD	Below LOD

*LOD* limit of detection

**Table 4:** Repeatability of the Cq values at the LOD for all *Listeria* SYBR®Green qPCR assays. The results are based on 36 repetitions with genomic targets as DNA template

	Repeatability calculation on *C*_q_ values at the LOD					
	*L. monocytogenes* 1/2a (ATCC 51772)		*L. monocytogenes* 4b (ATCC 51777)		*Listeria ivanovii* (LMG 11388)	
	RSDr (%)	*r*	RSDr (%)	*r*	RSDr (%)	*r*
*iap*-50-deg	4.9	4.8	na	na	2	1.9
*prs*-2-deg	2.4	2.2	na	na	2.3	2.1
*hlyA*-177	3.8	3.6	4.1	3.8	na	na
*hlyA*-146-deg-tronc	1.6	1.5	2.9	2.8	na	na

*na* not applicable, *LOD* limit of detection

**Table 5:** Repeatability of the Tm values for all *Listeria* SYBR®Green qPCR assays. The results are based on all Tm values coupled with amplification (Cq≠40)

	Repeatability calculation on *T*_m_ value								
	*L. monocytogenes* 1/2a (ATCC 51772)			*L. monocytogenes* 4b (ATCC 51777)			*Listeria ivanovii* (LMG 11388)		
	*T*_m_ value ± SD (°C)	RSDr (%)	*r*	*T*_m_ value ± SD (°C)	RSDr (%)	*r*	*T*_m_ value ± SD (°C)	RSDr (%)	*r*
*iap*-50-deg	76.8 ± 0.4	0.6	1.2	nt	na	na	75.9 ± 0.5	0.6	1.3
*prs*-2-deg	71.3 ± 0.2	0.2	0.5	nt	na	na	71.1 ± 0.3	0.4	0.7
*hlyA*-177	74.3 ± 0.2	0.3	0.6	73.7 ± 0.3	0.4	0.9	nt	na	na
*hlyA*-146-deg-tronc	74 ± 0.4	0.6	1.2	74.5 ± 0.3	0.4	0.9	nt	na	na

*nt* not tested, *na* not applicable

### Determination of reproducibility of SYBR®Green qPCR assays

The RSD_R_ was calculated for each sample on the *C*_q_ and the *T*_m_ values (Table 6). For each SYBR®Green qPCR assay, this RSD_R_ was between 0.05 and 0.95 % for the *T*_m_ values and was between 0.23 and 6.19 % for the *C*_q_ values. The RSD_R_ values of the developed SYBR®Green qPCR comply with the acceptance limits. The expanded uncertainty at 99 % of confidence has also been calculated from the reproducibility data from the eight samples. *U* was ranging between 0.46 and 1.42 °C for the *T*_m_ values and between 0.95 and 3.72 *C*_q_ for the *C*_q_ values (Table 6).

**Table 6:** Reproducibility test of the four SYBR®Green *Listeria* spp. and *L. monocytogenes* detection assays

		Sample	*L. mono* 1/2b (ATCC 51780)	*L. mono* 4d (BCRL: 7/89)	*L. mono* 4b (BCRL: 10/47)	*L. mono* 3b (Würburg)	*L. mono* 4a (ATCC 19114)	*L. mono* 1/2c (BCRL: 10/49)	*L. mono* 3a (BCRL: 9/109)	*L. mono* 4c (EU-RL: 09LEB41)	Uncertainty (99 %)
		Copy number	20	50	100	200	50	20	20	5	
*iap*-50-deg	*C*_q_	Average Lab 1	29.58	28.16	27.45	26.80	28.46	29.57	29.49	31.59	1.44
		Average Lab 2	29.15	27.37	27.65	26.67	27.29	28.45	28.52	32.03	
		RSD_R_ (%)	1.03	2.03	0.50	0.35	2.98	2.72	2.35	0.96	
	*T*_m_	Average Lab 1	77.00	77.00	76.50	76.75	76.50	77.00	77.00	76.50	1.21
		Average Lab 2	76.35	76.35	75.50	76.05	76.05	76.50	76.50	76.05	
		RSD_R_ (%)	0.60	0.60	0.93	0.65	0.42	0.46	0.46	0.42	
*prs-*2-deg	*C*_q_	Average Lab 1	28.38	26.62	26.04	25.44	27.31	28.36	28.45	30.03	0.95
		Average Lab 2	27.91	26.48	25.42	25.72	26.46	28.23	27.93	30.56	
		RSD_R_ (%)	1.19	0.35	1.70	0.78	2.24	0.35	1.31	1.24	
	*T*_m_	Average Lab 1	71.75	71.75	71.50	72.00	71.75	71.50	71.75	71.50	0.46
		Average Lab 2	71.60	71.60	71.45	71.45	71.60	71.60	71.45	71.45	
		RSD_R_ (%)	0.15	0.15	0.05	0.54	0.15	0.10	0.30	0.05	
*hlyA*-177	*C*_q_	Average Lab 1	30.28	28.33	27.51	29.32	28.83	30.05	29.57	31.88	1.30
		Average Lab 2	29.16	27.63	27.60	28.53	28.29	29.05	29.74	31.61	
		RSD_R_ (%)	2.66	1.77	0.23	1.92	1.33	2.41	0.40	0.61	
	*T*_m_	Average Lab 1	74.00	74.00	74.00	74.00	73.50	74.00	74.00	74.00	0.88
		Average Lab 2	73.75	73.75	73.40	73.40	73.20	73.75	73.40	73.40	
		RSD_R_ (%)	0.24	0.24	0.58	0.58	0.29	0.24	0.58	0.58	
*hlyA*-146-deg-tronc	*C*_q_	Average Lab 1	29.90	28.14	27.65	27.20	28.95	30.37	30.53	32.65	3.72
		Average Lab 2	27.65	26.03	26.23	25.70	26.75	28.46	27.96	31.28	
		RSD_R_ (%)	5.53	5.50	3.74	4.02	5.58	4.61	6.19	3.02	
	*T*_m_	Average Lab 1	75.00	75.00	74.75	75.00	73.50	73.75	74.00	74.75	1.42
		Average Lab 2	74.50	74.35	74.05	74.05	73.05	73.20	73.05	73.75	
		RSD_R_ (%)	0.47	0.62	0.67	0.90	0.43	0.53	0.91	0.95	

### Combination of the four SYBR®Green qPCR detection assays

Since the CoSYPS Path Food system is a screening (qualitative) system, the detection and discrimination of several *Listeria* species have been examined. The four assays have been run, on the same plate with the same PCR programme (as described previously), using the appropriate concentration of each primer (Table 2) on four gDNA extractions from pure cultures of *L. monocytogenes* serotype 4b, *L. ivanovii*, *L. seeligeri* and *L. grayi.* The four SYBR®Green qPCR assays amplified *L. monocytogenes* with a *C*_q_ value which is comparable between assays (Tables 7 and 8). The species *L. ivanovii* and *L. seeligeri* were amplified by the two assays amplifying the *Listeria* spp. The species *L. grayi* was amplified by none of the four assays (Tables 7 and 8).

**Table 7:** Matrix of targets amplification with the four SYBR®Green qPCR assays

Genus	Species	*iap*-50-deg	*prs-*2-deg	*hlyA*-177	*hlyA-*146-deg-tronc
*Listeria*	*monocytogenes*	+	+	+	+
*Listeria*	*ivanovii*	+	+	−	−
*Listeria*	*seeligeri*	+	+	−	−
*Listeria*	*welshimeri*	+	+	−	−
*Listeria*	*innocua*	+	+	−	−
*Listeria*	*grayi*	−	−	−	−

**Table 8:** Matrix of targets amplification with the four SYBR®Green qPCR assays: experimental verification

Sample Name	Detector	*iap*-50-deg		*prs*-2-deg		*hlyA*-177		*hlyA*-146-deg-tronc	
*L. monocytogenes* 4b	*C*_q_	23.45	22.99	21.66	21.57	22.95	22.75	22.48	22.58
	*T*_m_	76.9	76.9	71.7	71.7	74.1	74.1	74.7	74.7
	Conclusion	*Listeria* spp. present		*Listeria* spp. present		*L. monocytogenes* present		*L. monocytogenes* present	
	Average *C*_q_	22.55							
	St dev *C*_q_	0.65							
*L. ivanovii*	*C*_q_	23.69	23.29	22.23	22.11	Und	Und	34.67	38.67
	*T*_m_	75.9	76.3	71.7	71.7	72.6	74.7	74.7	71.7
	Conclusion	*Listeria* spp. present		*Listeria* spp. present		Below LOD		Below LOD	
	Average *C*_q_	22.83				na			
	St dev *C*_q_	0.78							
*L. seeligeri*	*C*_q_	26.73	26.09	25.02	25.48	34.72	37.91	Und	Und
	*T*_m_	75.9	75.9	71.7	71.7	74.1	74.1	73	72.1
	Conclusion	*Listeria* spp. present		*Listeria* spp. present		Below LOD		Below LOD	
	Average Cq	25.83				na			
	St dev *C*_q_	0.74							
*L. grayi*	*C*_q_	Und	35.02	34.17	Und	Und	35.86	Und	Und
	*T*_m_	70	74.4	71.5	71.7	74.4	74.4	70	70
	Conclusion	Below LOD		Below LOD		Below LOD		Below LOD	

## Discussion

*L. monocytogenes* is an important foodborne pathogen and is widely tested in food, environmental and clinical samples (Gasanov et al. 2005). The detection of *L. monocytogenes* is traditionally performed with culture methods using selective enrichment and plating, followed by characterization based on colony morphology and biochemical properties (Anonymous 1996a). Such methods are labour intensive and time consuming. Therefore, in the last decade, the need arose for the development of rapid detection methods (Postollec et al. 2011), especially in the case of bio-emergency. The development of PCR or qPCR assays in the field of microbiology has increased markedly and they are now generally accepted as a faster alternative to the conventional microbiological methods (Postollec et al. 2011). However, the success of a PCR or qPCR assays is based on the primer pair design and its efficient evaluation.

In this study, four qualitative SYBR®Green qPCR assays have been successfully developed to detect the presence of *Listeria* genus bacteria, as well as the specific identification of *L. monocytogenes*. The four SYBR®Green qPCR assays targets the *iap* and *hlyA* virulence genes and *prs*, a gene flanking a virulence cluster. The SYBR®Green qPCR strategy described is based on two detection levels. A first set of generic assays allows the detection of the presence of all bacteria belonging to the *Listeria* genus (except *L. grayi*). A second set of assays allows the specific detection of *L. monocytogenes*.

*L. grayi* is not detected by the first set of assays because *L. grayi* is a species that genetically differs significantly from the other species of the *Listeria* genus (e.g. 56 % amino acid homology for *iap* gene between *L. monocytogenes* and *L. grayi*) (Schmid et al. 2005). This was confirmed by the multiple alignment of the amplicons of *iap*-50-deg and *prs*-2-deg assays (data not shown). It is also important to note that the newly characterized species of *L. marthii* and *L. rocourtiae* strains have not been tested *in situ* since they were not available at the time of the experiments.

These qualitative SYBR®Green qPCR assays to detect *Listeria* spp. and discriminate *L. monocytogenes* were developed to be run simultaneously using a uniform PCR programme. Furthermore, in order to avoid any false negative detection due to sequence variation between strains, two SYBR®Green qPCR assays have been developed for each level of specificity: *Listeria* genus and *L. monocytogenes.* The SYBR®Green chemistry has been chosen for these qualitative detection assays since it is important to detect all strains. Indeed, with the TaqMan® technology, two primers and one probe should be designed instead of only two primers for the SYBR®Green technology. A higher homology of the amplicon sequence would then be required with TaqMan® technology. For instance, in the 37 nt length between the two primers of *iap-50-*deg assay, at least seven mismatches were observed among the different *Listeria* species (data not shown). Moreover, the SYBR®Green chemistry is less expensive than TaqMan® and allows a post-amplification verification of specificity of the amplicon (Postollec et al. 2011). All of these advantages make SYBR®Green technology the best choice for a screening system.

Most published PCR and qPCR assays to detect foodborne pathogens are not homogeneously validated (O’Grady et al. 2008; Oravcova et al. 2006; Rossmanith et al. 2006). ISO 22119 (Anonymous 2011c) gives the general definitions and requirements about the use of qPCR in food microbiology but does not give performance criteria and their acceptance limits to validate a qPCR assay. To date, the evaluation of the application of qPCR assay in food microbiology is performed according to ISO 16140 (Anonymous 2003), e.g. Rossmanith et al. (2006). This ISO 16140 norm gives the guidelines and acceptance criteria to compare an alternative method with the ISO reference method but does not allow the validation of the developed qPCR assays itself. In the present study, we propose a guideline to validate qPCR assays applied to food microbiology. All of the developed assays were evaluated for a set of performance criteria, specific to the qPCR applied to food microbiology. These performance criteria are not listed in an available guideline, so they were extracted from two available guidelines (Anonymous 2011b, 2008a). The first guideline is giving performance criteria to evaluate a PCR assay in food-microbiology (Anonymous 2011b), while the second (Anonymous 2008a) gives a list of performance criteria and their acceptance limits specific to the qPCR to evaluate a qPCR assay applied to genetically modified organisms (GMO) detection. Indeed, in the GMO field, qPCR is the golden standard for GMO detection in food and feed (Marmiroli et al. 2008). The evaluation of these performance criteria, according to these two guidelines, showed that the four developed SYBR®Green qPCR assays, *iap*-50-deg, *prs*-2-deg (for *Listeria* spp. (except *L. grayi*) detection) and *hlyA*-177, *hlyA*-146-deg-tronc (for *L. monocytogenes* detection), are highly accurate for their targets with 98.08 and 100 % accuracy, respectively. These assays are also very sensitive, with LOD between two and five copies per reaction (LOD should be between one to ten copies (Anonymous 2011b)). They are also efficient, with PCR efficiency between 97 and 107 %. They are repeatable, with RSD_r_ values far below the requested 25 %, and they are reproducible, with RSD_R_ values also far below the requested 35 %.

Besides the lack of performance criteria for qPCR in food microbiology, the qPCR assays to detect foodborne pathogens described so far are designed using different methodologies (classical PCR, real-time PCR using the TaqMan® or SYBR®Green chemistry) as well as different PCR programmes and protocols. This makes the simultaneous use of all of these methods impossible. However, such a simultaneous detection may turn out to be extremely useful when a more global approach is necessary or when a rapid identification of the foodborne pathogens is requested in a bio-emergency or outbreaks of unknown origin.

The four SYBR®Green assays were developed to be run using the same PCR programme. They can be assembled on a single plate in order to save time and reduce plate-to-plate variation. The combination of these four assays, based on two levels of detection, results in a high-quality screening system and a remarkable food surveillance tool. This qPCR system will give an answer on the presence/absence of *Listeria* ssp. in the sample and will at the same time indicate if the detected *Listeria* is *L. monocytogenes* which is the strain mandatory in EU regulation 2073/2005 (Anonymous 2005). The detection of other *Listeria* species may be useful to uncover other origins of contamination by the *Listeria* genus as few cases of listeriosis have been attributed to *L. ivanovii* (Cummins et al. 1994; Guillet et al. 2010; Lessing et al. 1994), *L. innocua* (Perrin et al. 2003) and *L. seeligeri* (Rocourt et al. 1986). Moreover, the post-amplification dissociation curve, a tool inherent to SYBR®Green chemistry, will give information about the specificity of the amplicon, further reducing false positive conclusions as well as giving information about the species or the serotypes amplified (Table 2). A similar qPCR detection system that detects both *L. monocytogenes* and the other *Listeria* species simultaneously is already available (Pall GeneDisc® *Listeria* DUO) (http://www.pall.be/pdfs/Biopharmaceuticals/nexidia_listeria_id_genesystems_aoac_2009-v2.pdf). However, contrary to the CoSYPS Path Food system, this commercial kit is not modular. Indeed the CoSYPS Path Food system could be easily adapted to target other important foodborne pathogens such as *Salmonella* spp., *Campylobacter* spp. or verotoxin-producing *Escherichia coli* using the same screening platform. The detection assays will be added to or removed from the screening system in function of the bacteria sought in the sample. A future study will focus on the validation of the present qPCR system compared with the ISO reference method (microbiological methods) (Anonymous 1996a) on food samples according to ISO 16140 (Anonymous 2003).
